# Surgical management of bifocal femoral fractures: a systematic review and pooled analysis of treatment with a single implant versus double implants

**DOI:** 10.1007/s00402-023-04950-7

**Published:** 2023-07-05

**Authors:** J. D. Cnossen, Esther M. M. Van Lieshout, Michael H. J. Verhofstad

**Affiliations:** Trauma Research Unit, Department of Surgery, Erasmus MC, University Medical Center Rotterdam, P.O. Box 2040, 3000 CA Rotterdam, The Netherlands

**Keywords:** Femoral shaft fractures, Bifocal fractures, Proximal femur, Intramedullary nailing

## Abstract

**Introduction:**

Fractures of the proximal femur accompanied by a fracture of the femoral shaft are relatively rare, with a reported prevalence between 1 and 12%. Multiple surgical options are available, consisting of treatment with a single implant or with double implants. Controversy exists about the optimal management. A systematic review and pooled analysis were performed to assess the most reliable treatment for bifocal femoral fractures of the femur.

**Materials and methods:**

A literature search was conducted on July 15, 2022. Selected studies were screened on title and abstract by two researchers independently, and full texts were read by both authors. Emphasis was put on adverse events such as postoperative infection, healing complications, malalignment, and functional outcome using either a single implant or double implants.

**Results:**

For the proximal femoral fractures, no significant difference could be confirmed for avascular necrosis of the femoral neck (5.1% for single implant and 3.8% for double implants), nonunion (6.4% for single implant and 7.8% for double implants), or varus malalignment (6.6% for single implant and 10.9% for double implants). This study also suggests that the number of implants is irrelevant for complications of the femoral shaft regarding the rates of postoperative infection and healing complications. Pooled rates of bone healing complications were 1.6–2.7-fold higher when patients were treated with a single implant, but statistical significance could not be confirmed. For hardware failure, revision surgery, leg length discrepancy, and functional outcome, no difference between the two groups was found either.

**Conclusions:**

The pooled proportions of all postoperative complications had overlapping confidence intervals; thus, no inference about a statistically significant difference on the number of implants used for treating ipsilateral fractures of the femur can be made. Both treatment groups showed a similar functional outcome at the last moment of follow-up, with more than 75% of the patients reporting a good outcome.

**Supplementary Information:**

The online version contains supplementary material available at 10.1007/s00402-023-04950-7.

## Introduction

Bifocal fractures of the femur have first been reported in the early fifties [1, 2]. Fractures of the femoral neck or trochanteric fractures accompanied by a fracture of the femoral shaft are rare. The reported prevalence of these bifocal fractures ranges between 1 and 12% [3–9]. These injuries are usually the result of high-energy trauma (HET) and occur mostly in polytraumatized young adults [10]. AO-type A3, AO-type B, and AO-type C femoral shaft fracture patterns are more often part of a bifocal injury [11].

Due to the extent of trauma, femoral neck fractures are easily missed during the primary survey. Previous literature reports that up to 30% of femoral neck fractures are missed [10, 12], and increased awareness showed a decline in missed fractures [9, 13]. In bifocal fractures, the femoral shaft fracture is usually accompanied by a fracture of the femoral neck, but up to 28% is accompanied by a trochanteric fracture [11, 14, 15].

Besides diagnostic challenges, the management of bifocal fractures of the femur can be challenging as well. To regain function, anatomic reduction of the femoral neck fracture, restoring the length and alignment of the femoral shaft fracture, and create a stable osteosynthesis should be the goal of initial surgical management. Since the first reports various treatment options that are developed, literature reports up to 50 possible treatment options for bifocal fractures of the femur. These treatment modalities can be divided into two groups, namely management with a single implant or with a separate implant for each fracture, referred to as double implants. Treatment with a single implant allows the physician to treat both fractures with one single device showing various results [16–22]. The alternative is using separate implants for each fracture [9, 12, 23–29]. Several authors recommend to prioritize fixation of the femoral neck to prevent further risk of the blood supply of the femoral head [30, 31].

Controversy still exists about the optimal management of bifocal femur fractures. Using a single implant or double implants has shown good results, although mostly in small numbers [19, 20, 27, 32–35]. A systematic review and pooled analysis was performed to assess the outcome in bifocal fractures of the femur on adverse events such as postoperative infection, healing complications, malalignment, and functional outcome using either on the two types of treatment.

## Materials and method

### Literature search strategy

This systematic review with pooled analysis was conducted following the Preferred Reporting Items for Systematic Reviews and Meta-Analyses criteria [36]. With the help of a biomedical information specialist, a literature search was completed on July 15, 2022. Embase, Medline Ovid, Cinahl, Web of Science, Cochrane, and Google scholar were used in the search. The databases were searched on terms related to “femoral shaft fractures” combined with “proximal femur fracture”, “femoral neck fracture” and their abbreviations and synonyms. The search strings used for each database are shown in Appendix 1 of the supplemental material.

Inclusion criteria were studies that described adult patients that were treated operatively for combined acute fractures of the femoral shaft and the proximal femur using either a single implant or with double implants. Fractures of the proximal femur included fractures of the femoral neck and trochanteric fractures. Studies that did not publish about primary treatment (e.g., treatment of delayed or nonunion) or studies that reported non-original data (e.g., systematic reviews or meta-analysis), case series, or biomechanical studies were excluded. Studies published before 2000 were excluded as well to remove the older generation of intramedullary nails; therefore, emphasis was put on most recent generations of intramedullary nails. Duplicate studies were removed.

First, selected studies were screened on title and abstract by two researchers independently (JDC and EMMVL). Second, full texts were read by both authors. In both screenings, a consensus was reached by discussion. When any inconsistencies remained after discussion, the third author (MHJV) was consulted. All inconsistencies were resolved by consensus. If a full text was not available, the corresponding author was contacted by the first author (JDC) by e-mail. Data from studies not published in English were extracted with the help of a narrative speaker of the language of the publication.

### Data extraction

Data were extracted by two authors (JDC and EMMVL) independently, using a predefined data sheet. The following data were collected: (1) general information and demographic information (i.e., number of patients, number of fractures, sex, and age), (2) injury characteristics (i.e., trauma mechanism, associated injuries, and injury severity score), (3) treatment characteristics (i.e., operation time, blood loss during operation, number of patients treated with a single implant, and number of patients treated with a double implant), and (4) clinical outcome (i.e., adverse events, revision surgery, and functional outcome).

Patients treated with a single implant were considered a distinct group, and patients treated with a double implant were considered a distinct group. A single implant treatment was defined as one implant treating both injuries. In treatment with a double implant, each fracture is fixated with a separate device (e.g. cannulated hip screws or sliding hip screw for the femoral neck fracture and retrograde nailing for the femoral shaft fracture, or antegrade intramedullary nailing with cannulated hip screws).

### Assessment of quality selected studies

The quality of each included study was assessed using the methodologic index for nonrandomized studies (MINORS), which is a validated instrument for nonrandomized surgical studies [37]. Seven items are selected for assessing non-comparative studies, and additionally five items are selected for assessing of comparative studies. Ideally non-comparative studies can score 16 points and comparative studies 24 points. Patients were stratified into a group with patients treated with a single implant for both fractures and a group with patients in whom the fractures were treated with a separate implant (double implant group). The quality of the studies is shown in Appendix 2 of the supplemental material.

### Analysis

Data were analyzed using MedCalc Statistical Software version 18.2.1 (MedCalc Software bvba, Ostend, Belgium; http:/www.medcalc.org; 2018). Pooled estimates were reported with their 95% confidence intervals (CI). Cochran’s Q-test and the *I*^2^ were used to quantify the heterogeneity. For the pooled analysis, a fixed effects model was used when the *I*^2^ was lower than 40%, and a random effects model was used when the *I*^2^ was higher than 40%. For each outcome, forest plots and funnel plots were used to assess publication bias. The funnel plots showed no substantial anomalies that raised the suspicion of a publication bias. The forest plots of each outcome are shown in Appendix 3, and the funnel plots are shown in Appendix 4 of the supplemental material. Numeric data of the forest plots are shown in Tables 2, 3 and 4.

## Results

**Fig. 1:**
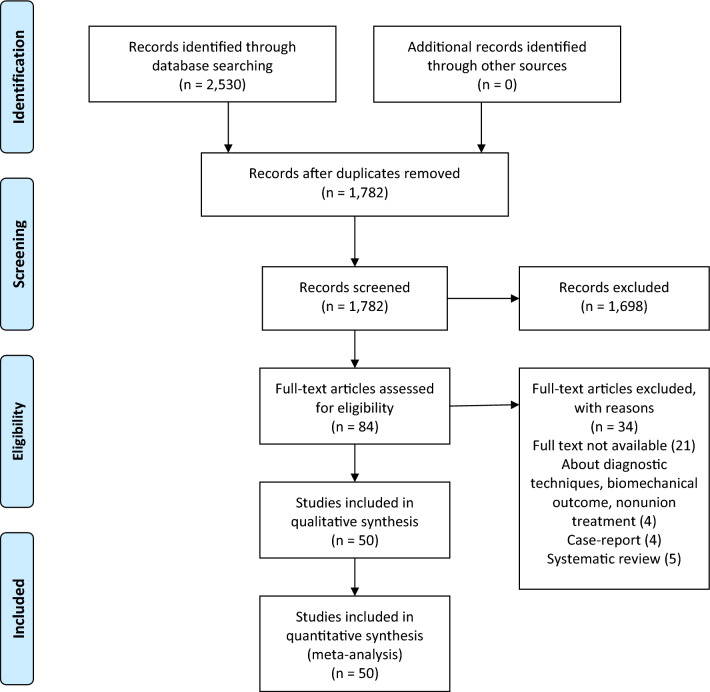
Inclusion flowchart

A total of 2530 studies were identified with the search strategy (Fig. 1). After de-duplication and screening the title and abstract of all records, 84 studies remained for full-text analysis. After assessing full text, 34 articles were excluded based on the exclusion criteria. In total, 50 articles (1310 patients) were included in the final analysis [3–9, 12, 18–21, 23–29, 32–35, 38–64]. There were six prospective studies and 44 retrospective articles. General data for all studies are presented in Table 1. The majority of the patients were male and sustained a concomitant fracture of the femur as result of high-energy trauma. Of the patients, 625 were treated with a single implant and 660 were treated with a double implant. Mean follow-up ranged from 12 to 78 months.

**Table 1:** Overview of included studies

Author (year)	Study period	Patients (fractures)	Male patients	Mean Age (range)	HET	Proximal fractures included^a^	Missed^b^	S.I.	D.I.	Time to union PF (months)	Time to union SF (months)	Mean follow-up (range)
Chaudhary et al. (2021) [61]	2018–2020	7 (7)	5 (71%)	35 (27–45)	7 (100%)	FN + TF	N.A.	0	7	4.1 (3–6)	5.1 (3–11)	16.2 (10–22)
Rana et al. (2021) [62]	2018–2020	13 (13)	8 (62%)	38 (23–54)	13 (100%)	FN	N.A.	13	0	4.3 (N.A.)	N.A.	17 (9–25)
Wei et al. (2021) [63]	2003–2019	22 (22)	15 (68%)	45 (20–80)	N.A.	FN	N.A.	6	16	N.A.	N.A.	12 (N.A.)
Singh et al. (2021) [64]	2017–2019	17 (17)	13 (76%)	35 (27–55)	17 (100%)	FN + TF	N.A	0	17	5.1 (3–7)	6.5 (4–9)	15.1 (12–18)
Angelini et al. (2021) [60]	January 2015–December 2018	12 (12)	7 (58%)	53 (24–90)	12 (100%)	FN + TF	0 (0%)	6	6	N. A	N.A.	16.2 (6–26)
Xiang et al. (2021) [59]	January 2015 to September 2018	33 (33)	24 (72%)	44 (18–72)	33 (100%)	FN	N.A	11	22	5.6 (–)	4.9 (–)	N.A
Kang et al. (2020) [28]	January 2012–December 2016	14 (14)	10 (71%)	35 (17–60)	14 (100%)	FN + TF	0 (0%)	14	0	4.2 (3–19)	5 (3–17)	17.3 (9–30)
Oh et al. (2020) [29]	October 2015 and June 2018	10 (10)	10 (100%)	47 (24–75)	10 (100%)	FN	0 (0%)	0	10	4.0 (12–20)	6.2 (18–40)	20.5 (14–46)
Shin et al. (2020) [58]	March 2013–December 2018	28 (28)	20 (71%)	54 (24–73)	N.A	TF	1 (4%)	28	0	3.3 (–)	5.0 (–)	N.A.
Spitler et al. (2020) [18]	N.A	26 (26)	18 (69%)	40 (16–73)	26 (100%)	FN	0 (0%)	26	0	N.A	N.A.	22.4 (3.5–95.1)
Lawson et al. (2017) [35]	January 2005–June 2015	10 (10)	7 (70%)	46 (29–62)	10 (100%)	FN + TF	0 (0%)	5	5	5.1 (3–12)	5 (3–8)	43.5 (6–108)
Zhao et al. (2016) [19]	October 2012 to January 2016	10 (10)	8 (80%)	36 (18–50)	10 (100%)	FN	0 (0%)	0	10	3.1 (2.7–4.1)	5.1 (4.1–5.5)	18.0 (12–24)
von Rüden et al. (2015) [57]	2004–2013	65 (65)	47 (72%)	45 (19–90)	65 (100%)	FN	2 (20%)	36	29	N.A.	N.A.	N.A.
Ostrum et al. (2014) [9]	May 2002–October 2011	92 (92)	70 (76%)	33 (17–83)	N.A	FN + TF	6 (7%)	0	92	N.A	N.A.	23.9 (4–72)
Park et al. (2014) [56]	August 1995 and January 2012	26 (26)	21 (81%)	40 (16–69)	26 (100%)	FN + TF	6 (23%)	0	26	3.3 (1.8–4.6)	5.6 (3.7–9.2)	26.1 (12–72)
Bali et al. (2013) [32]	July 2009 to December 2010	16 (16)	10 (63%)	42 (31–59)	N.A	FN + TF	1 (6%)	16	0	N.A	N.A	N.A
Gadegone et al. (2013) [55]	December 2005–December 2011	36 (36)	29 (81%)	39 (28–64)	26 (72%)	FN + TF	2 (6%)	36	0	4.8 (4–8)	6.2 (6–9)	12 (12–12)
Habib et al. (2012) [34]	January 2007–March 2011	13 (13)	10 (77%)	31 (19–45)	11 (84%)	FN	0 (0%)	0	13	3.2 (–)	4.4 (3.7–8.3)	18 (12–36)
Kesemenli et al. (2012) [27]	1995–2005	41 (41)	32 (78%)	34 (21–53)	41 (100%)	FN + TF	8 (19%)	17	24	3.7 (3.2–5.3)	7.1 (3.7–10.6)	N.A.
Wang et al. (2012) [54]	March 2004–April 2009	23 (23)	19 (83%)	N.A	23 (100%)	FN + TF	0 (0%)	10	13	3.9 (2.8–4.6)	5.0 (4.6–8.3)	17.4 (12–48)
Gary et al. (2011) [33]	2001–2006	22 (23)	19 (86%)	38 (19–66)	N.A	FN	5 (22%)	10	13	N.A	N.A	N.A.
Tsarouhas et al. (2011) [20]	January 2004–December 2008	11 (11)	11 (100%)	46 (18–75)	11 (100%)	FN	0 (0%)	11	0	4.5 (4–6)	7.2 (6–9)	47 (15–75)
Douša et al. (2010) [21]	November 1994–December 2008	66 (66)	56 (85%)	41.8 (–)	63 (96%)	FN + TF	N.A	66	0	N.A.	N.A.	N.A.
Neto et al. (2010) [52]	August 2002 and October 2007	17 (17)	15 (88%)	33 (19–47)	17 (100%)	FN + TF	1 (6%)	8	9	N.A.	N.A.	48.5 (17–79)
Wang et al. (2010) [53]	January 2004–May 2008	21 (21)	18 (86%)	42 (25–60)	21 (100%)	FN	1 (5%)	10	11	3.6 (2.8–4.6)	4.8 (3.7–7.3)	24.5 (12–48)
Bedi et al. (2009) [26]	1989–2006	37 (37)	18 (49%)	38 (18–73)	N.A	FN	0 (0%)	9	28	N.A.	N.A.	34.4 (12–112)
Cannada et al. (2009) [12]	2001–2005	89 (91)	51 (57%)	36 (15–72)	89 (100%)	FN	24 (26%)	19	72	3.8 (–)	3.8 (–)	17 (2–54)
Tsai M et al. (2009) [50]	January 2000–January 2008	43 (43)	28 (65%)	43 (17–73)	43 (100%)	FN	6 (14%)	5	38	3.5 (2–5)	6.5 (3–12)	48 (6–70)
Tsai C et al. (2009) [25]	January 1999–December 2005	37 (37)	21 (57%)	42 (18–70)	37 (100%)	FN	N.A.	37	0	3.7 (1.4–6.8)	8.3 (5.7–10.3)	23 (12–45)
Vidyadhara et al. (2009) [7]	January 1997–January 2004	43 (43)	32 (74%)	43 (29–55)	43 (100%)	FN	N.A.	43	0	3.9 (2.2–7.1)	6 (2.8–11.7)	49.4 (25–81)
Abalo et al. (2008) [5]	April 1997–September 2004	37 (37)	29 (78%)	37 (18–69)	37 (100%)	FN + TF	N.A.	0	37	4 (2–5)	6 (4–8)	49 (22–82)
Peskun et al. (2008) [6]	1993–2003	26 (26)	16 (62%)	44 (20–76)	22 (85%)	FN + TF	N.A.	13	13	N.A.	N.A.	49.6 (13–120)
Schmal et al. (2008) [8]	January 2001–July 2007	21 (22)	18 (86%)	43 (–)	N.A	FN + TF	0 (0%)	17	5	N.A.	N.A.	N.A
Singh et al. (2008) [49]	January 2000–December 2006	27 (27)	24 (89%)	35 (–)	27 (100%)	FN	0 (0%)	12	15	3.7 (–)	4.9 (–)	25.5 (19–34)
Wang et al. (2008) [51]	January 2000–August 2005	20 (20)	12 (60%)	45 (19–76)	18 (90%)	FN	2 (10%)	1	18	N.A.	N.A	15 (5–48)
Oh et al. (2007) [47]	Oktober 1998–september 2005	33 (33)	N.A	N.A	33 (100%)	FN + TF	7 (21%)	16	17	N.A.	N.A	33 (19–68)
Shetty and Kumar (2007) [48]	January 1995–January 2005	34 (34)	30 (88%)	35 (18–60)	32 (94%)	FN + TF	7 (21%)	27	7	3.0 (2.3–5.4)	4.1 (3.2–7.8)	28 (1–5)
Kao et al. (2006) [45]	January 1999–December 2003	15 (15)	7 (45%)	45 (21–70)	15 (100%)	FN	0 (0%)	15	0	3.5 (1.5–7.0)	8.7 (6–11)	23.7 (13–45)
Oh et al. (2006) [46]	October 2000–June 2004	16 (17)	14 (88%)	44 (25–60)	16 (100%)	FN + TF	1 (6%)	0	17	2.5 (1.8–2.7)	6.3 (3.3–13.8)	N.A.
Kakkar et al. (2005) [43]	May 1999–April 2002	7 (7)	N.A	N.A	7 (100%)	FN	0 (0%)	7	0	3.7 (3.2–4.6)	4.6 (3.7–5.1)	N.A.
Khallaf et al. (2005) [24]	March 1996–June 2002	17 (17)	15 (88%)	37 (20–60)	17 (100%)	FN	0 (0%)	2	15	4.0 (2.5–5)	5.4 (4–9)	43.2 (24–72)
Pavelka et al. (2005) [44]	January 1998–February 2003	19 (19)	14 (74%)	48 (19–79)	15 (79%)	FN + TF	N.A.	19	0	N.A.	N.A.	N.A.
Daglar et al. (2004) [42]	March 1999–Jan 2002	19 (19)	15 (79%)	26 (18–41)	19 (100%)	FN + TF	N.A.	0	19	N.A.	3.5 (2.5–8)	22.5 (12–33)
Hung et al. (2004) [23]	July 1982 to July 1998	42 (42)	34 (81%)	36 (16–67)	42 (100%)	FN + TF	N.A.	17	25	N.A.	N.A.	56.4 (26–147)
Jain et al. (2004) [4]	January 1998–December 2001	23 (23)	22 (96%)	35 (21–56)	23 (100%)	FN + TF	2 (9%)	21	2	4.4 (3.2–9.6)	5 (3.2–7.3)	24.1 (12–47)
Barei et al. (2003) [41]	January 1996–May 2001	7 (7)	5 (71%)	43 (19–63)	7 (100%)	FN	2 (29%)	1	6	N.A.	N.A.	19.3 (4–56)
Okcu and Aktuglu (2003) [3]	January 1990 to December 1998	15 (15)	14 (93%)	36 (22–57)	15 (100%)	FN	4 (27%)	0	15	4.8 (SD 0.8)	6.1 (SD 2.1)	78 (52–150)
Lin et al. (2002) [40]	February 1999 to March 2001	5 (5)	4 (80%)	41 (29–59)	5 (100%)	FN	1 (20%)	5	0	10.5 (5.7–13.5)	N.A.	12.9 (5.7–17.3)
Chen et al. (2001) [39]	August 1989–February 1998	18 (18)	14 (78%)	40 (22–77)	16 (89%)	FN	2 (11%)	1	17	4.2 (SD 2.0)	5.2 (SD 2.3)	41 (9–105)
Elshafie et al. (2001) [38]	April 1992–January 1998	9 (9)	9 (100%)	29 (18–36)	9 (100%)	FN	1 (11%)	9	0	4.2 (3–6)	6.9 (4–18)	25.2 12–36

*HET* high energy trauma, *S.I.* single implants, *D.I.* double implants, *Time to union PF* time to union of the proximal fracture, *Time to union SF* time to union of the femoral shaft fracture, *FN* femoral neck fracture, *PF* peritrochanteric fracture, *N.A.* not available or not described in text, *SD* standard deviation

^a^Proximal fractures included, are the type of proximal fractures that were included in the study

^b^Number of missed fractures described by the article. Age is displayed in number of years, other time related data is displayed in months (time to union PF, time to union SF, mean time follow up)

### Operation time and perioperative blood loss

Only seven studies reported sufficient data concerning operation time and perioperative blood loss [19, 28, 49, 54, 59, 61, 62]. Table 2 shows the pooled analysis for operation time and perioperative blood loss for both treatment groups. It also shows the number of studies, the number of patients for which data were available, and the result of the heterogeneity test. The pooled operation time for single implants was 133 min. (95% CI 98–169 min.) versus 150 min. (95% CI 124–177 min.) for patients treated with a double implant [19, 28, 49, 54, 59, 61, 62]. The overlapping confidence intervals suggest that operation time is unrelated to the number of implants.

**Table 2:** Operative time and perioperative blood loss in treatment with a single implant or a double implant

Parameter	Studies	Patients	Cochran’s *Q* (*p*-value)	*I*^2^ (95% CI)	Method	Pooled mean (95% CI)
Operation time (min)						
S.I.	5	62	472.8 (< 0.001)	99.2% (98.8–99.4%)	Random	133 min (98–169)
D.I.	5	67	87.6 (< 0.001)	95.4% (91.9—97.4%)	Random	150 min (124–177)
Blood loss (mL)						
S.I.	4	48	448.5 (< 0.001)	99.3% (99.1—99.5%)	Random	334 mL (152–516)
D.I.	4	52	146.2 (< 0.001)	98.0% (96.6–98.8%)	Random	373 mL (233–512)

*CI* confidence interval, *D.I.* double implant, *N.A.* not available, *mL* milliliters, *min* minutes, *S.I.* single implant

In the group treated with a single implant, pooled perioperative blood loss was 334 mL (95% CI 152–516 mL) compared to 373 mL (95% CI 233–512 mL) in patients treated with a double implant [19, 54, 59, 61, 62].

### Adverse events of the femoral neck fractures

In patients treated with a single implant, postoperative infection was seen in 6.1% (95% CI 1.1–14.4%) of the patients (Table 3) [21, 38, 55]. Insufficient data were available concerning postoperative infection in patients treated with a double implant. Most avascular necrosis (AVN) was found in the single implant group with 5.1% (95% CI 2.8–8.5%) of the patients versus 3.8% (95% CI 1.5–7.2%) in patients treated with a double implant [4, 7, 9, 12, 18, 21, 25, 46, 49, 55, 59, 62, 63]. The overlapping confidence intervals do not suggest evidence of a difference between the type of treatment and the risk of AVN. Nonunion of the femur neck was noted in 6.4% (95% CI 3.9–9.4%) when treated with a single implant and in 8.2% (95% CI 5.3–11.6%) when treated with a separate device for each fracture [3–5, 7–9, 12, 18, 21, 25, 26, 28, 32, 33, 43, 45–50, 55, 56, 61]. Varus malunion was noted in 6.6% of the patients in the single implant group (95% CI 3.9–10.1%) and in 10.9% in the double implant group (95% CI 6.6–16.2%) [3–5, 7, 9, 21, 24–27, 38, 41, 42, 44, 46, 49, 52, 55, 57, 61, 63]. The pooled rates were higher for the double implant group but the overlapping confidence intervals suggest that nonunion and varus malunion of the femur neck are unrelated to the number of implants used.

**Table 3:** Adverse events of the proximal femur, the femur shaft, general adverse events, and the rate of revision surgery after a bifocal fracture of the femur

Parameter	Studies	Patients	Cochran’s *Q* (*p*-value)	*I*^2^ (95% CI)	Method	Pooled portion (95% CI)
Adverse events femoral neck						
Infection						
S.I.	3	111	3.8 (0.1476)	48% (0–85%)	Random	6.1 (1.1–14.4%)
D.I.	0	0	N.A	N.A	N.A	N.A
AVN						
S.I.	9	250	4.2 (0.8359)	0% (0–34%)	Fixed	5.1 (2.8–8.5%)
D.I.	6	242	6.4 (0.2711)	21.6% (0–66%)	Fixed	3.8 (1.5–7.2%)
Nonunion						
S.I.	13	328	11.1 (0.5202)	0% (0–53%)	Fixed	6.4 (3.9–9.4%)
D.I.	13	374	14.4 (0.2749)	16.8 (0–56%)	Fixed	8.2 (5.3–11.6%)
Varus malunion						
S.I.	10	234	7.2 (0.6122)	0% (0–53%)	Fixed	6.6 (3.9–10.1%)
D.I.	13	324	21.9 (0.0389)	45% (0–71%)	Random	10.9 (6.6–16.2%)
Adverse events femoral shaft						
Infection						
S.I.	7	177	7.5 (0.2795)	19.7% (0–63%)	Fixed	5.8 (2.9–10.2%)
D.I.	12	301	19.4 (0.0544)	43% (0–71%)	Random	9.2 (5.2–14.3%)
Delayed union						
S.I.	9	202	35.6 (< 0.001)	77.5% (57–88)	Random	19.9 (9.3–33.3%)
D.I.	10	231	9.4 (0.3998)	4.4% (0–64%)	Fixed	10.8 (7.1–15.4%)
Nonunion						
S.I.	20	363	53.3 (< 0.001)	64% (43–78%)	Random	17.3 (11.1–24.6%)
D.I.	14	430	41.6 (< 0.001)	66% (42–81%)	Random	14.2 (8.8–20.7%)
Malunion						
S.I.	2	20	0.6 (0.4410)	0% (0–0%)	Fixed	17.5 (4.9–35.7%)
D.I.	4	164	8.0 (0.0462)	63% (0–87%)	Random	6.4 (1.3–15.0%)
General adverse events						
Hardware failure						
S.I.	8	182	18.1 (0.0116)	61.3% (16–82%)	Random	11.4 (4.9–20.2%)
D.I.	5	96	1.6 (0.8145)	0% (0–50%)	Fixed	9.9 (4.9–17.5%)
Revision surgery						
S.I.	16	345	46.4 (< 0.001)	67.7% (46–81%)	Random	18.4 (11.6–26.4%)
D.I.	13	276	15.0 (0.2405)	20.1% (0–58%)	Fixed	17.0 (12.2–22.5%)

*AVN* avascular necrosis, *CI* confidence interval, *D.I.* double implant, *N.A.* not available, *S.I*. single implant

### Adverse events of the femoral shaft fractures

Higher rates of infection were found when treated with double implants 9.2% (95% CI 5.2–14.3%) versus 5.8% (95% CI 2.9–10.2%) when treated with a single implant [4, 21, 27, 28, 54, 55, 61, 62, 64]. The overlap in 95% confidence intervals does not suggest a superior treatment. In all bone healing-related complications, treatment with a single implant showed 1.6–2.7-fold higher pooled rates compared to patients treated with double implants, which is, respectively, 19.9% (95% CI 9.3–33.3%) in single implants versus 10.8% (95% CI 7.1–15.4%) in double implants for delayed union [4, 6, 7, 9, 21, 27, 38, 46, 49, 52, 53, 56, 61, 62, 64]. Nonunion was seen in 17.3% (95% CI 11.1–24.6%) in single implant versus 14.2% (95% CI 8.8–20.7%) in double implants [4, 5, 9, 12, 18, 20, 21, 23–25, 27, 29, 33, 34, 39, 43–45, 47, 50, 53–58, 60, 61, 63]. Malunion was seen in 17.5% (95% CI 4.9–35.7%) when treated with a single implant versus 6.4% (95% CI 1.3–15.0%) in patients treated with double implants [9, 20, 25, 26, 52, 57]. However, the overlapping confidence intervals showed no convincing evidence of a statistical difference between the two groups.

### General adverse events

Hardware failure was noted in 11.4% (95% CI 4.9–20.2%) of patients treated with a single implant versus 9.9% (95% CI 4.9–17.5%) in patients treated with a double implant [4, 7, 20, 23, 24, 27, 42, 45, 46, 50, 53, 57]. The reoperation rate was 18.4% (95% CI 11.6–26.4%) versus 17.0% (95% CI 12.2–22.5%), respectively, for single implants and for double implants [3, 7, 9, 18–21, 24, 27, 32, 34, 38, 41, 42, 44, 45, 47, 49, 50, 53–56]. The overlapping confidence intervals suggested no relationship between these complications and the number of implants.

### Functional outcome

Leg length discrepancy was found in 12.4% (95% CI 8.5–17.3%) of the patients treated with a single implant and in 8.8% (95% CI 2.9–17.4%) of the patients treated with a double implant [4, 7, 20, 27, 38, 44–46, 49, 50, 53, 55, 56] (Table 4).

**Table 4:** Functional outcome after treatment with a single implant or a double implant

Parameter	Studies	Patients	Cochran’s *Q* (*p*-value)	*I*^2^ (95% CI)	Method	Pooled portion (95% CI)
LLD						
S.I.	11	230	6.8 (0.7454)	0% (0–42%)	Fixed	12.4 (8.5–17.3%)
D.I.	3	54	1.9 (0.3886)	0% (0–96%)	Fixed	8.8 (2.9–17.4%)
FW-score (good)						
S.I.	14	211	24.9 (0.0236)	48% (3–72%)	Random	75.9 (67.2–83.6%)
D.I.	19	347	31.4 (0.0262)	43% (1–67%)	Random	77.0 (70.9–82.6%)

*CI* confidence interval, *D.I.* double implant, *FW-score (good)* Friedman and Wyman score displayed as proportion of patients scoring a “good” score, *LLD* leg length discrepancy, *S.I.* single implant

The Friedman and Wyman criteria were used in most studies as a tool to measure the functional outcome [65]. Patients treated with a single implant showed a good outcome according to the Friedman and Wyman criteria in 75.9% (95% CI 67.2–83.6%) versus 77.0% (95% CI 70.9–82.6%) for patients treated with double implants [3, 5, 23–25, 27–29, 34, 35, 39, 40, 46–50, 53–57, 64]. Overlapping confidence intervals suggesting the number of implants are irrelevant to the functional outcome.

## Discussion

Concomitant fractures of the proximal femur and the femoral shaft present a difficult management problem for the physician. Controversy exist about the number of implants that should be used and which fracture should be prioritized in stabilization. Therefore, a pooled analysis was conducted to analyze the effect of the number of implant used and the amount of adverse events and the functional outcome. This systematic review and pooled analysis found overlapping confidence intervals for complications after surgery for concomitant fractures of the femur. Regarding the femoral neck, no significant difference could be confirmed for postoperative infection, avascular necrosis of the femoral neck, nonunion, or varus malalignment. This study could not confirm the superiority of either single of double implant with respect to the risk of postoperative infection and healing complications. The same was true for hardware failure, revision surgery, leg length discrepancy, and functional outcome.

The primary goal of the treatment should be based on: (1) adequate anatomic reduction of the proximal femur fracture and the femoral shaft fracture; (2) restore alignment, length, and rotation of the femur; and (3) provide a stable construct for both fractures. Achieving this will reduce the risk of complications. Multiple combination implant options are available each with their own advantages. In the early nineties, the indication for the use of reconstruction nails extended to concomitant fractures of the femur with good results [66–68]. Some studies suggest that reconstruction nailing has the advantages of a minimal invasive technique, reduced blood loss, and shorter operation time [4, 25, 53, 54, 67]. This pooled analysis showed that this is not necessarily the case with single implants.

Well-known complications of treatment for proximal femur fractures are avascular necrosis, nonunion, and varus malunion. Reported rates of avascular necrosis of the neck range from 0 to 9% in the literature [9, 21, 30, 69]. The hypothesis is that higher rates of avascular necrosis in single implants are expected due to the displacement of the femoral neck when the antegrade nail is inserted [66, 69, 70]. This displacement when rodding the femoral nail could increase the risk of impairment of the blood circulation of the femoral head [31, 70, 71]. This raised the question on which fracture should be stabilized first. The current pooled analysis showed pooled proportions of 3.8% of avascular necrosis when treated with double implants and 5.1% of avascular necrosis of the femoral head when treated with a single implant, which are similar rates in patients with isolated femoral neck fractures [72–74].

This study showed comparable nonunion rates of the femoral neck in both treatment groups, respectively, 6.4% for the single implant group and 7.8% when treated with double implants. A prompt anatomic reduction is the best treatment to reduce nonunion rate. The union rate may decrease where the stability of the fixation has been jeopardized [26, 33, 75–78]. Watson et al*.* reported that 7 of 8 patients with femoral neck nonunion had significant varus malunion [79]. An accurate reduction is more often accomplished in patients treated with retrograde nailing for the shaft and a separate implant for the neck compared with a cephalomedullary device [26]. Another potential benefit of dual implants lies in the management of complications. The complications of shaft fractures can often be managed without taking out femoral neck fixation when patients are treated with separate devices.

Although the pooled proportions were determined per treatment group and the overlap in confidence intervals, for avascular, necrosis and nonunion does not confirm superiority of either one of the treatment options, the authors prefer to prioritize the neck fracture since avascular necrosis or inadequate reduction can lead to devastation outcome in this relative young population.

### Shaft complications

This pooled analysis showed 1.6–2.7-fold higher pooled proportions of healing complications in the patients treated with a single implant for both fractures; however, the overlapping confidence intervals suggesting the number of implants are irrelevant to bone-healing complications. The rate of shaft complications in bifocal fractures is higher than in isolated femoral shaft fractures; this could be explained by the larger amount of energy which is transferred trough the femur during trauma. [3, 4, 7, 33, 45, 49]. Compared with isolated femoral shaft fractures, higher rates of healing complications in bifocal fractures are reported for the femoral shaft [80–85]. In the data of this study, we could not derive any contributing factors to nonunion of the femoral shaft, mostly to the lack of studies reporting injury characteristics (such as open fractures) or postoperative instructions. Postoperative factors such as type of implant (especially the type of generation implant), or postoperative weight bearing could be a contributing factor to nonunion. In the studies in this pooled analysis, no difference was observed in the type of implant used, especially the generation of the implants, in nonunion. For postoperative weight bearing, more research is needed, only a few studies reported on postoperative weight bearing after the treatment on bifocal femoral fractures. Previous studies did show factors contributing to nonunion of the femoral shaft; open shaft fractures, unreamed nails, malreduction, prolonged weight bearing, and the use of reconstruction nails [26, 79]. This could explain the higher rates of healing complications for the shaft, but more data are needed as nonunion is the major concern in these types of fractures.

### Functional outcome

The functional outcome after concomitant fractures of the proximal femoral and the femoral shaft is good in the majority of patients. This study showed that 75.9% of the patients treated with a single implant showed a good score on the Friedman and Wyman score versus 77.0% for the group treated with double implants. Although frequently used as an instrument to report functional outcome, the Friedman and Wyman score is a non-validated tool to assess functional outcome. However, in other (non-)validated instruments, the functional outcome appears to be good as well [6, 7, 19, 20, 44, 50, 59, 86–88].

### Limitations

This study has several limitations, which warrants careful interpretation of the findings. First, almost only retrospective studies were included. No randomized controlled trials were retrieved with this search string. Because of the large amount of case series, a comparative meta-analysis between the two groups was impossible; thus, a pooled analysis was conducted. Second, relatively small numbers of studies on certain outcomes were published and the continuous data were not always provided (range, mean, standard deviation, or number of patients). This gives insufficient data in certain domains. Despite these limitations, the strength of this study is the high number of studies included in the analysis (providing data of 50 studies and 1310 patients).

## Conclusion

This systematic review and pooled analysis showed that treatment with a single implant and double implant is both suitable options for concomitant fractures of the proximal femur and femur shaft. However, 1.6–2.7-fold higher pooled proportions of healing complications in patients treated with a single implant treating both fractures were observed. All pooled proportions had overlapping confidence intervals, suggesting that currently available data do not confirm a statistically significant association between postoperative complications and the number of implants used for treating ipsilateral fractures of the femur. Both treatment groups showed similar functional outcome at the last moment of follow-up, with still twenty-five percent of the patients reporting bad outcome regardless of the treatment used.

## Supplementary Information

Below is the link to the electronic supplementary material.

Supplementary file1 (DOCX 16 KB)

Supplementary file2 (DOCX 16 KB)

Supplementary file3 (DOCX 884 KB)

Supplementary file4 (DOCX 708 KB)

## Data Availability

Data will be made available upon reasonable request to the corresponding author.
