# Supplementary material for: Surgical management of bifocal femoral fractures: a systematic review and pooled analysis of treatment with a single implant versus double implants
Source: Arch Orthop Trauma Surg. 2023 Jul 5;143(10):6229–41. doi: 10.1007/s00402-023-04950-7 (PMC10491515; doi:10.1007/s00402-023-04950-7)
Supplement: Supplementary file 1 — Supplementary file1 (DOCX 16 KB) [file 402_2023_4950_MOESM1_ESM.docx]

**Appendix 1: search terms used for databases: femoral shaft fracture & femoral neck fracture**

**Embase.com**

('femur shaft fracture'/de OR ('femoral shaft'/de AND 'femur fracture'/de) OR (((femur* OR femoral*) NEAR/3 (shaft* OR diaphys*) NEAR/6 (fracture*))):ab,ti,kw) AND ('femoral neck fracture'/de OR 'femur intertrochanteric fracture'/de OR 'femur pertrochanteric fracture'/de OR ('femoral neck'/de AND ('femur fracture'/de OR 'proximal femur fracture'/de)) OR (((femur* OR femoral*) NEAR/3 (neck*) NEAR/6 (fracture*)) OR ((intertrochanteric* OR pertrochanteric*) NEAR/3 fracture*)):ab,ti,kw) OR (((ipsilateral* NEAR/6 fracture*):ab,ti,kw) AND ('femur fracture'/de OR ((femur* OR femoral*) NEAR/6 fracture*):ti))

**Medline Ovid**

((((femur* OR femoral*) ADJ3 (shaft* OR diaphys*) ADJ6 (fracture*))).ab,ti,kw.) AND (Femoral Neck Fractures/ OR (Femur Neck/ AND exp Femoral Fractures/) OR (((femur* OR femoral*) ADJ3 (neck*) ADJ6 (fracture*)) OR ((intertrochanteric* OR pertrochanteric*) ADJ3 fracture*)).ab,ti,kw.) OR (((ipsilateral* ADJ6 fracture*).ab,ti,kw.) AND (Femoral Fractures/ OR ((femur* OR femoral*) ADJ6 fracture*).ti.))

**Cinahl EBSCOhost**

(TI((((femur* OR femoral*) N2 (shaft* OR diaphys*) N5 (fracture*)))) OR AB((((femur* OR femoral*) N2 (shaft* OR diaphys*) N5 (fracture*))))) AND ((MH "Femur Neck" AND MH "Femoral Fractures"+) OR TI(((femur* OR femoral*) N2 (neck*) N5 (fracture*)) OR ((intertrochanteric* OR pertrochanteric*) N2 fracture*)) OR AB(((femur* OR femoral*) N2 (neck*) N5 (fracture*)) OR ((intertrochanteric* OR pertrochanteric*) N2 fracture*))) OR ((TI(ipsilateral* N6 fracture*) OR AB(ipsilateral* N6 fracture*)) AND (MH "Femoral Fractures" OR TI((femur* OR femoral*) N6 fracture*)))

**Web of Science Core Collection**

TS=(((((femur* OR femoral*) NEAR/2 (shaft* OR diaphys*) NEAR/5 (fracture*)))) AND ((((femur* OR femoral*) NEAR/2 (neck*) NEAR/5 (fracture*)) OR ((intertrochanteric* OR pertrochanteric*) NEAR/2 fracture*)))) AND TI=(((femur* OR femoral*) NEAR/5 (fracture*)))

**Cochrane Central Register of Controlled Trials**

((((femur* OR femoral*) NEAR/3 (shaft* OR diaphys*) NEAR/6 (fracture*))):ab,ti,kw) AND ((((femur* OR femoral*) NEAR/3 (neck*) NEAR/6 (fracture*)) OR ((intertrochanteric* OR pertrochanteric*) NEAR/3 fracture*)):ab,ti,kw) OR (((ipsilateral* NEAR/6 fracture*):ab,ti,kw) AND (((femur* OR femoral*) NEAR/6 fracture*):ti))

**Google scholar (top 200)**

"femur|femoral shaft|diaphysis|diaphyseal fracture" "femur|femoral neck fracture"|"intertrochanteric|pertrochanteric fracture" intitle:"femur|femoral fracture"
