# Supplementary material for: Surgical management of bifocal femoral fractures: a systematic review and pooled analysis of treatment with a single implant versus double implants
Source: Arch Orthop Trauma Surg. 2023 Jul 5;143(10):6229–41. doi: 10.1007/s00402-023-04950-7 (PMC10491515; doi:10.1007/s00402-023-04950-7)
Supplement: Supplementary file 2 — Supplementary file2 (DOCX 16 KB) [file 402_2023_4950_MOESM2_ESM.docx]

**Table 2: MINOR appraisal scores for the included studies**

| **Author (year)** | **Clearly stated aim** | **Inclusion of consecutive patients** | **Prospective collection of data** | **Endpoint appropriate to the study aim** | **Unbiased evaluation of endpoints** | **Follow-up period appropriate to endpoint** | **Loss to follow up not**  **exceeding 5%** | **Prospective calculation of study size** | **A control group having the gold standard intervention** | **Contemporary groups** | **Baseline equivalence of groups** | **Statistical analyses adapted to study design** | **Total** |
| --- | --- | --- | --- | --- | --- | --- | --- | --- | --- | --- | --- | --- | --- |
| Chaudhary et al. (2021) | 2 | 2 | 2 | 2 | 0 | 2 | 0 | 0 | N.A. | N.A. | N.A. | N.A. | 10/16 |
| Rana et al. (2021) | 2 | 2 | 1 | 1 | 0 | 2 | 1 | 0 | N.A. | N.A. | N.A. | N.A. | 9/16 |
| Wei et al. (2021) | 2 | 2 | 1 | 2 | 0 | 2 | 1 | 0 | N.A. | N.A. | N.A. | N.A. | 10/16 |
| Singh et al. (2021) | 1 | 2 | 2 | 2 | 0 | 2 | 2 | 0 | N.A. | N.A. | N.A. | N.A. | 11/16 |
| Angelini et al. (2021) | 2 | 2 | 1 | 2 | 0 | 2 | 1 | 0 | N.A. | N.A. | N.A. | N.A. | 10/16 |
| Xiang et al. (2021) | 2 | 2 | 1 | 2 | 1 | 0 | 2 | 0 | N.A. | N.A. | N.A. | N.A. | 10/16 |
| Kang et al. (2020) | 2 | 2 | 1 | 1 | 2 | 2 | 0 | 0 | N.A. | N.A. | N.A. | N.A. | 10/16 |
| Oh et al. (2020) | 2 | 2 | 1 | 2 | 0 | 2 | 2 | 0 | N.A. | N.A. | N.A. | N.A. | 11/16 |
| Shin et al. (2020) | 2 | 2 | 1 | 2 | 0 | 2 | 1 | 0 | N.A. | N.A. | N.A. | N.A. | 10/16 |
| Spitler et al. (2020) | 2 | 2 | 1 | 2 | 0 | 1 | 1 | 0 | N.A. | N.A. | N.A. | N.A. | 9/16 |
| Lawson et al. (2017) | 2 | 2 | 1 | 2 | 0 | 1 | 2 | 0 | N.A. | N.A. | N.A. | N.A. | 10/16 |
| Zhao et al. (2016) | 1 | 2 | 1 | 2 | 0 | 2 | 1 | 0 | N.A. | N.A. | N.A. | N.A. | 9/16 |
| von Rüden et al. (2015) | 0 | 2 | 1 | 2 | 0 | 2 | 2 | 0 | N.A. | N.A. | N.A. | N.A. | 9/16 |
| Ostrum et al. (2014) | 2 | 2 | 1 | 2 | 0 | 1 | 2 | 0 | N.A. | N.A. | N.A. | N.A. | 10/16 |
| Park et al. (2014) | 2 | 2 | 1 | 2 | 0 | 2 | 2 | 0 | N.A. | N.A. | N.A. | N.A. | 11/16 |
| Bali et al. (2013) | 2 | 2 | 1 | 2 | 0 | 2 | 2 | 0 | N.A. | N.A. | N.A. | N.A. | 11/16 |
| Gadegone et al. (2013) | 2 | 2 | 2 | 2 | 0 | 1 | 2 | 0 | N.A. | N.A. | N.A. | N.A. | 11/16 |
| Habib et al. (2012) | 1 | 2 | 1 | 2 | 0 | 2 | 2 | 0 | N.A. | N.A. | N.A. | N.A. | 10/16 |
| Kesemenli et al. (2012) | 2 | 2 | 1 | 2 | 0 | 2 | 2 | 0 | 0 | 1 | 0 | 2 | 14/24 |
| Wang et al. (2012) | 2 | 2 | 1 | 2 | 0 | 2 | 2 | 0 | 1 | 1 | 2 | 2 | 17/24 |
| Gary et al. (2011) | 2 | 2 | 1 | 2 | 0 | 2 | 2 | 0 | N.A. | N.A. | N.A. | N.A. | 11/16 |
| Tsarouhas et al. (2011) | 2 | 2 | 1 | 2 | 0 | 2 | 2 | 0 | N.A. | N.A. | N.A. | N.A. | 11/16 |
| Douša et al. (2010) | 2 | 2 | 1 | 1 | 0 | 2 | 2 | 0 | N.A. | N.A. | N.A. | N.A. | 10/16 |
| Neto et al. (2010) | 2 | 2 | 1 | 2 | 0 | 2 | 2 | 0 | N.A. | N.A. | N.A. | N.A. | 11/16 |
| Wang et al. (2010) | 2 | 2 | 1 | 2 | 0 | 2 | 2 | 0 | 1 | 1 | 2 | 2 | 17/24 |
| Bedi et al. (2009) | 2 | 2 | 1 | 2 | 0 | 2 | 2 | 0 | 1 | 1 | 0 | 2 | 15/24 |
| Cannada et al. (2009) | 2 | 2 | 1 | 2 | 0 | 1 | 1 | 0 | N.A. | N.A. | N.A. | N.A. | 9/16 |
| Tsai M et al. (2009) | 1 | 2 | 1 | 2 | 0 | 2 | 2 | 0 | N.A. | N.A. | N.A. | N.A. | 10/16 |
| Tsai C et al. (2009) | 2 | 2 | 1 | 2 | 0 | 1 | 2 | 0 | 0 | 1 | 2 | 2 | 15/24 |
| Vidyadhara et al. (2009) | 1 | 2 | 2 | 1 | 0 | 2 | 1 | 0 | 2 | 2 | 2 | 2 | 17/24 |
| Abalo et al. (2008) | 0 | 2 | 0 | 2 | 0 | 2 | 2 | 0 | N.A. | N.A. | N.A. | N.A. | 8/16 |
| Peskun et al. (2008) | 2 | 2 | 1 | 2 | 0 | 2 | 1 | 0 | 2 | 2 | 2 | 2 | 18/24 |
| Schmal et al. (2008) | 2 | 2 | 1 | 1 | 0 | 1 | 2 | 0 | N.A. | N.A. | N.A. | N.A. | 9/16 |
| Singh et al. (2008) | 2 | 2 | 1 | 2 | 0 | 2 | 2 | 0 | 0 | 2 | 2 | 2 | 17/24 |
| Wang et al. (2008) | 2 | 2 | 1 | 0 | 0 | 1 | 2 | 0 | N.A. | N.A. | N.A. | N.A. | 8/16 |
| Oh et al. (2007) | 2 | 2 | 1 | 2 | 0 | 2 | 2 | 0 | 0 | 2 | 0 | 2 | 15/24 |
| Shetty et al. (2007) | 0 | 2 | 1 | 2 | 0 | 2 | 2 | 0 | N.A. | N.A. | N.A. | N.A. | 9/16 |
| Kao et al. (2006) | 2 | 2 | 1 | 2 | 0 | 2 | 1 | 0 | N.A. | N.A. | N.A. | N.A. | 10/16 |
| Oh et al. (2006) | 2 | 2 | 1 | 2 | 0 | 1 | 1 | 0 | N.A. | N.A. | N.A. | N.A. | 9/16 |
| Kakkar et al. (2005) | 1 | 2 | 0 | 1 | 0 | 2 | 2 | 0 | N.A. | N.A. | N.A. | N.A. | 8/16 |
| Khallaf et al. (2005) | 2 | 2 | 1 | 2 | 0 | 2 | 2 | 0 | N.A. | N.A. | N.A. | N.A. | 11/16 |
| Pavelka et al. (2005) | 2 | 2 | 1 | 2 | 0 | 2 | 1 | 0 | N.A. | N.A. | N.A. | N.A. | 10/16 |
| Dağlar et al. (2004) | 2 | 2 | 1 | 2 | 0 | 2 | 2 | 0 | 0 | 2 | 0 | 2 | 15/24 |
| Hung et al. (2004) | 2 | 2 | 1 | 0 | 0 | 2 | 2 | 0 | N.A. | N.A. | N.A. | N.A. | 9/16 |
| Jain et al. (2004) | 2 | 2 | 1 | 2 | 0 | 2 | 2 | 0 | N.A. | N.A. | N.A. | N.A. | 11/16 |
| Barei et al. (2003) | 2 | 2 | 1 | 2 | 0 | 1 | 2 | 0 | N.A. | N.A. | N.A. | N.A. | 10/16 |
| Okcu et al. (2003) | 2 | 2 | 1 | 2 | 1 | 2 | 1 | 0 | N.A. | N.A. | N.A. | N.A. | 11/16 |
| Lin et al. (2002) | 2 | 2 | 1 | 2 | 0 | 1 | 2 | 0 | N.A. | N.A. | N.A. | N.A. | 10/16 |
| Chen et al. (2001) | 1 | 2 | 1 | 1 | 0 | 1 | 2 | 0 | N.A. | N.A. | N.A. | N.A. | 8/16 |
| Elshafie et al. (2001) | 1 | 2 | 1 | 1 | 0 | 2 | 2 | 0 | N.A. | N.A. | N.A. | N.A. | 9/16 |

N.A.; not applicable. The items are scored as “0” (not reported), “1” (reported but inadequate), or “2” (reported and adequate). The global ideal score being 16 for non-comparative studies and 24 for comparative studies.
