# Supplementary material for: Surgical management of bifocal femoral fractures: a systematic review and pooled analysis of treatment with a single implant versus double implants
Source: Arch Orthop Trauma Surg. 2023 Jul 5;143(10):6229–41. doi: 10.1007/s00402-023-04950-7 (PMC10491515; doi:10.1007/s00402-023-04950-7)
Supplement: Supplementary file 3 — Supplementary file3 (DOCX 884 KB) [file 402_2023_4950_MOESM3_ESM.docx]

**Figure 2: Forest plots of the pooled analyses of complications of the femur neck. Forest plots for single implant.**

|  | **Single implant** | **Double implant** |
| --- | --- | --- |
| **AVN** | 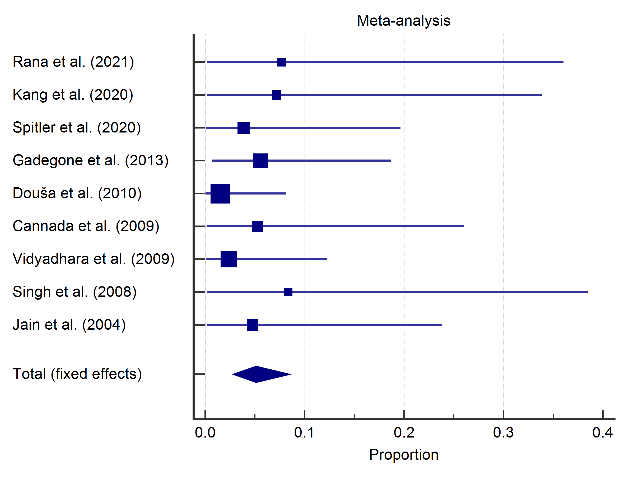 | 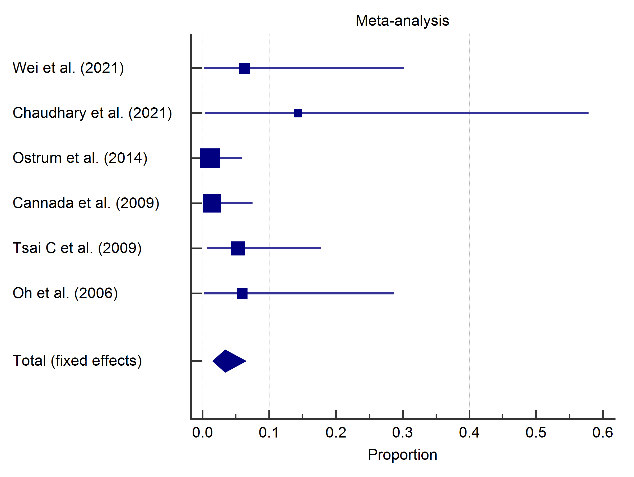 |
| **Nonunion** | **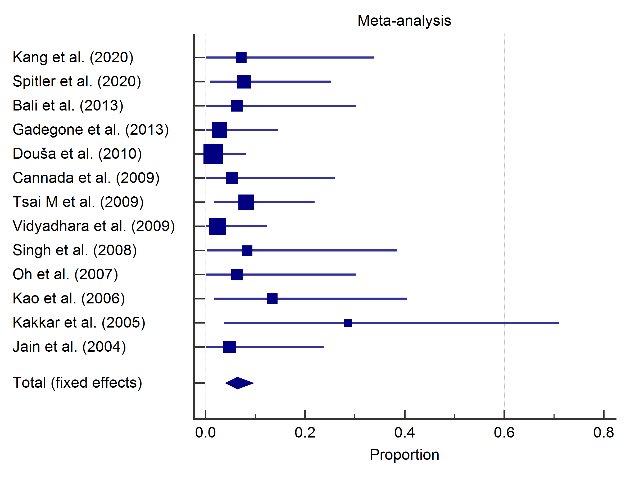** | **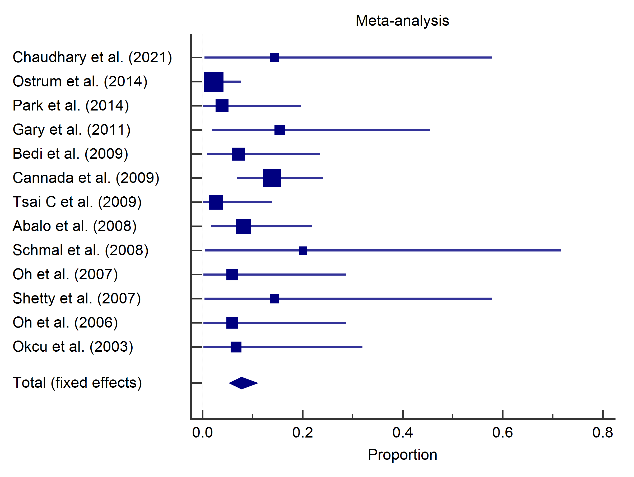** |
| **Varus malalignment** | **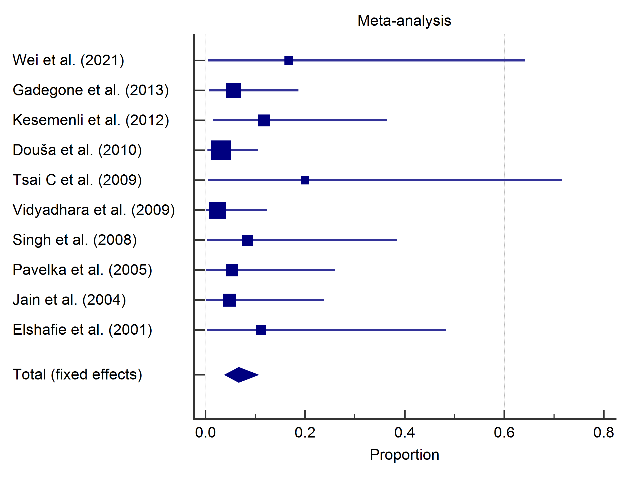** | **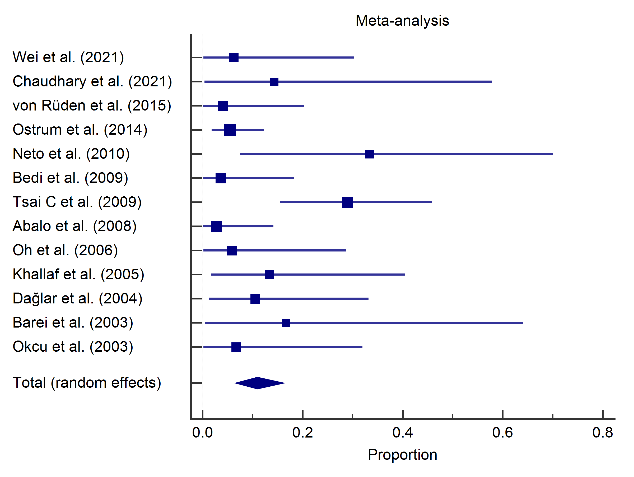** |

**Figure 3: Forest plots of the pooled analyses of the complications of the femoral shaft. Forest plots for single implant are displayed and the left and for double implants on the right side.**

**are displayed and the left and for double implants on the right side.**

|  | **Single implant** | **Double implant** |
| --- | --- | --- |
| **Postoperative infection** | **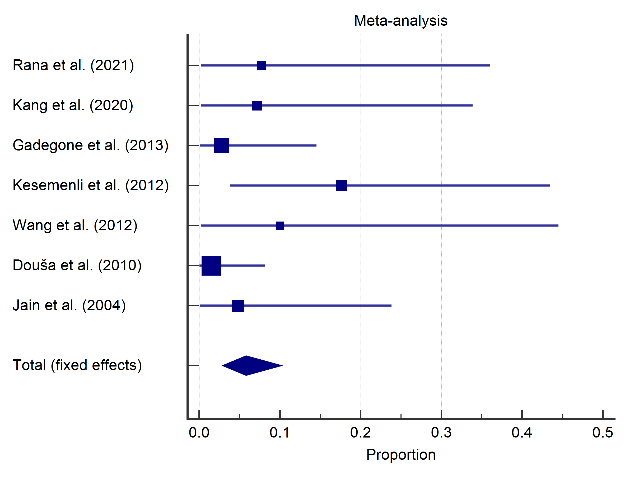** | 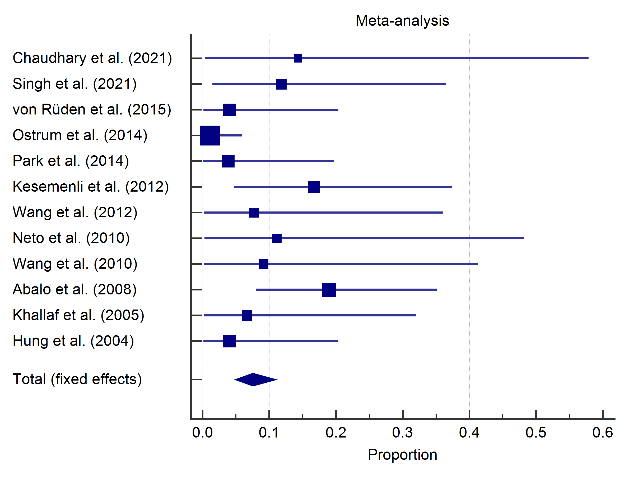 |
| **Delayed union** | 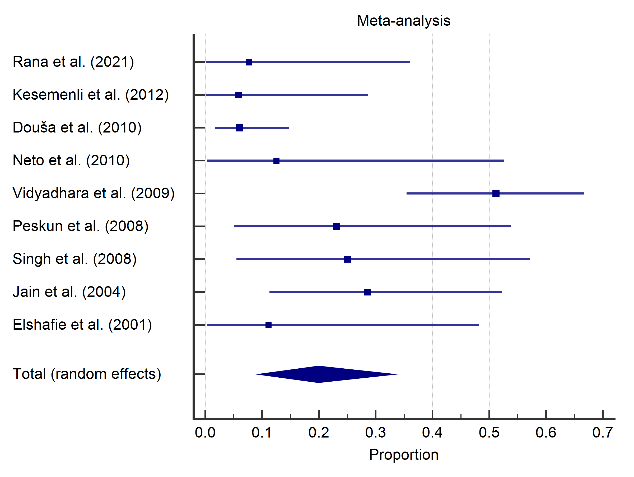 | 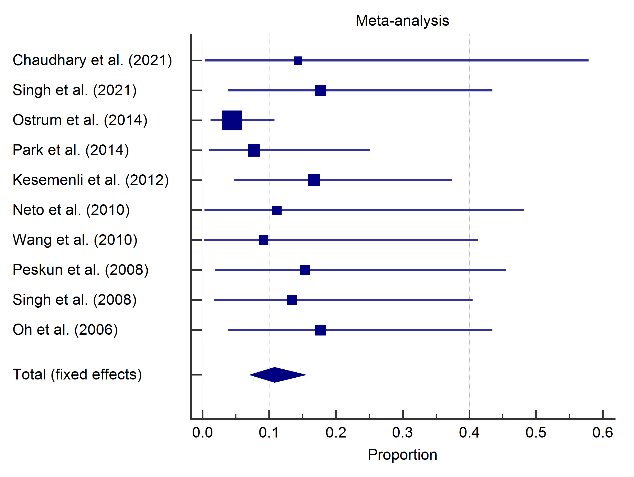 |
| **Nonunion** | **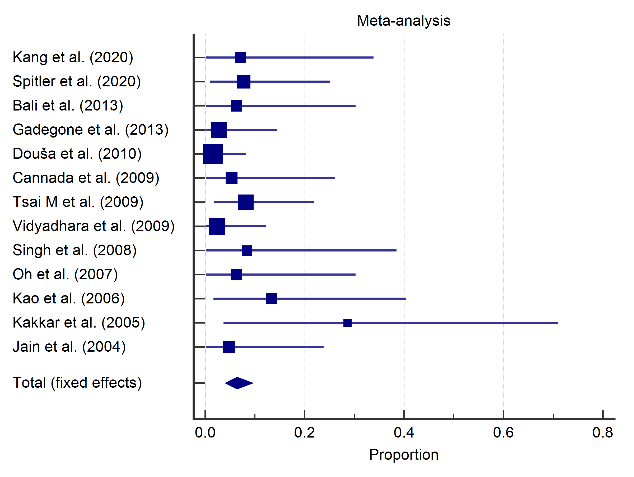** | **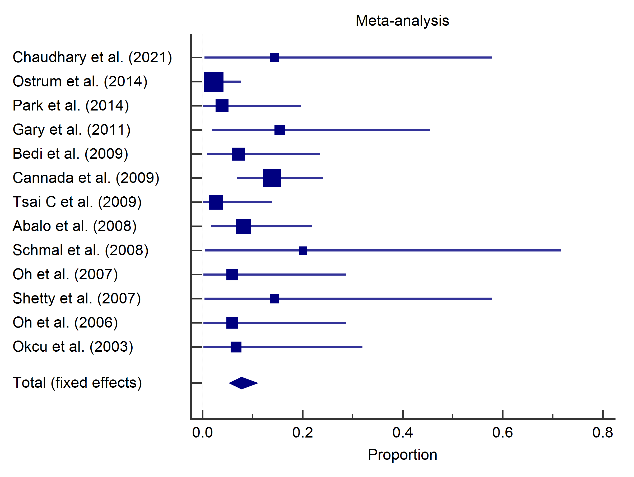** |
| **Malunion** | 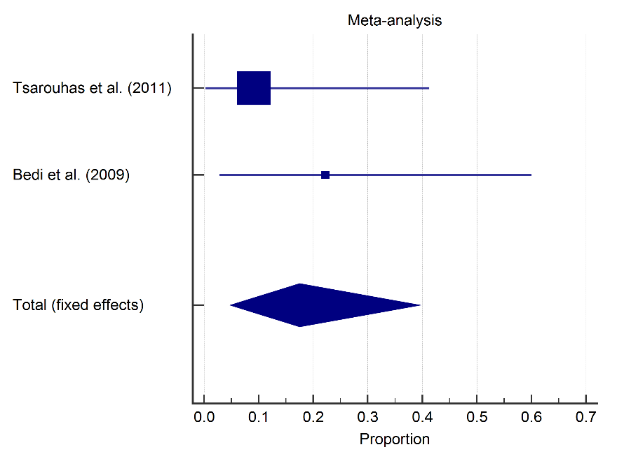 | 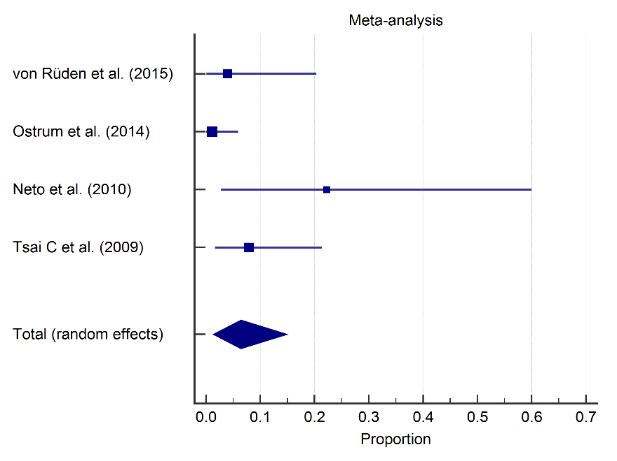 |

**Figure 4: Forest plots of the pooled analyses of the hardware failure and revision surgery. Forest plots for single implant are displayed and the left and for double implants on the right side.**

| **Hardware failure** | 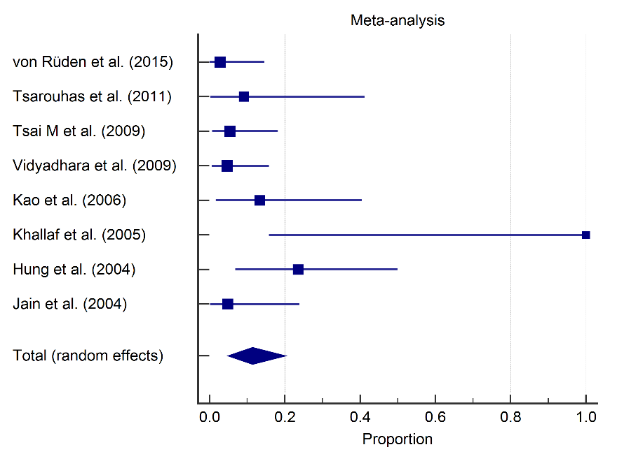 | 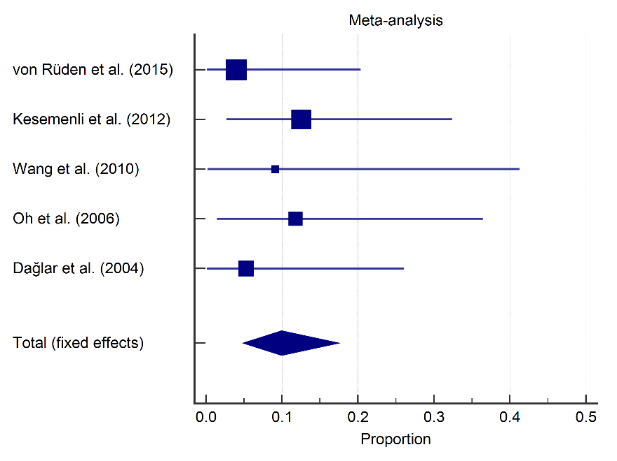 |
| --- | --- | --- |
| **Revision surgery** | 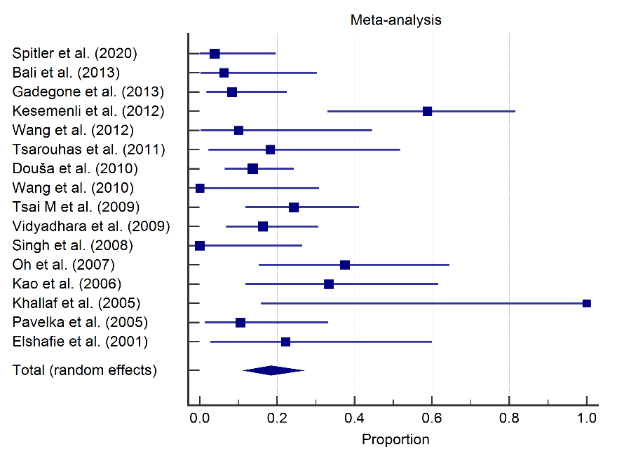 | 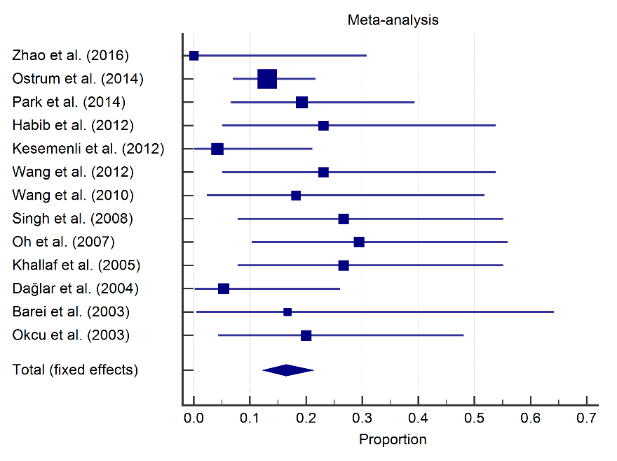 |

**Figure 5: Forest plots of the pooled analyses of the functional outcome. Forest plots for single implant are displayed and the left and for double implants on the right side.**

| **Leg length discrepancy** | 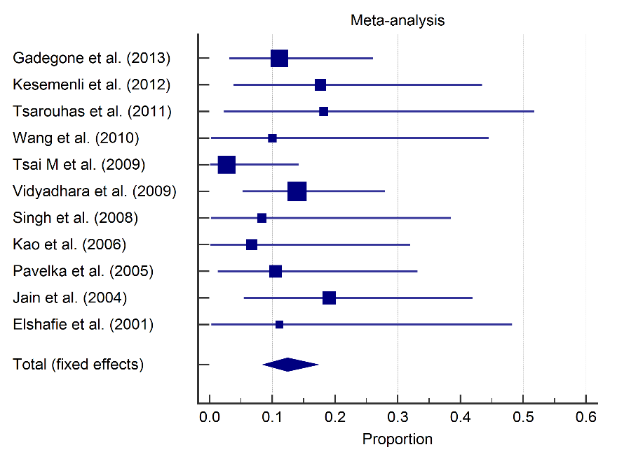 | 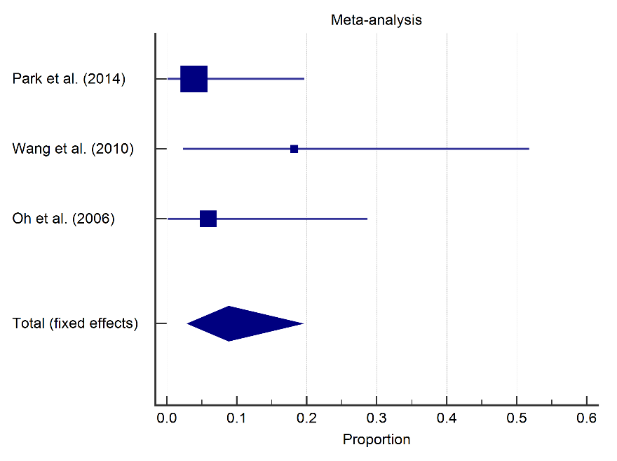 |
| --- | --- | --- |
| **Functional outcome** | 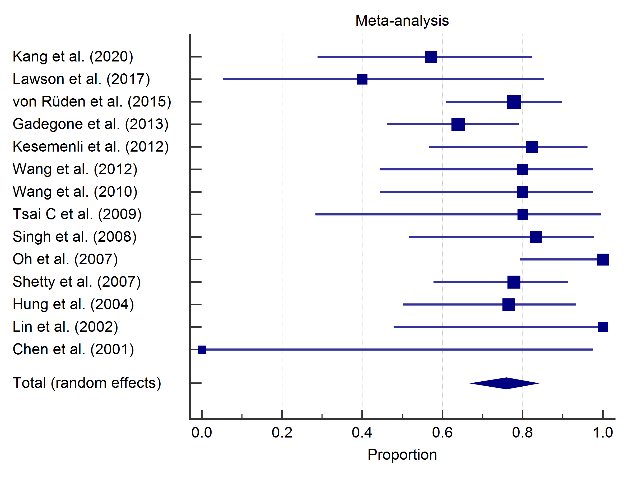 | 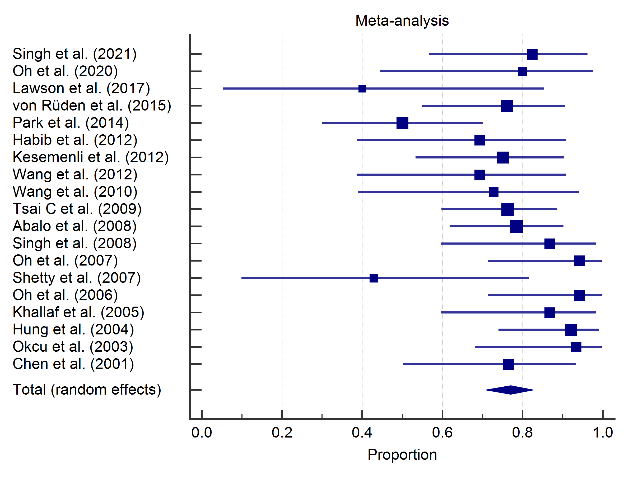 |
