# Supplementary material for: Surgical management of bifocal femoral fractures: a systematic review and pooled analysis of treatment with a single implant versus double implants
Source: Arch Orthop Trauma Surg. 2023 Jul 5;143(10):6229–41. doi: 10.1007/s00402-023-04950-7 (PMC10491515; doi:10.1007/s00402-023-04950-7)
Supplement: Supplementary file 4 — Supplementary file4 (DOCX 708 KB) [file 402_2023_4950_MOESM4_ESM.docx]

**Figure 6: Funnel plots of the pooled analyses of complications of the femur neck. Funnel plots for single implant are displayed and the left and for double implants on the right side.**

|  | **Single implant** | **Double implant** |
| --- | --- | --- |
| **AVN** | **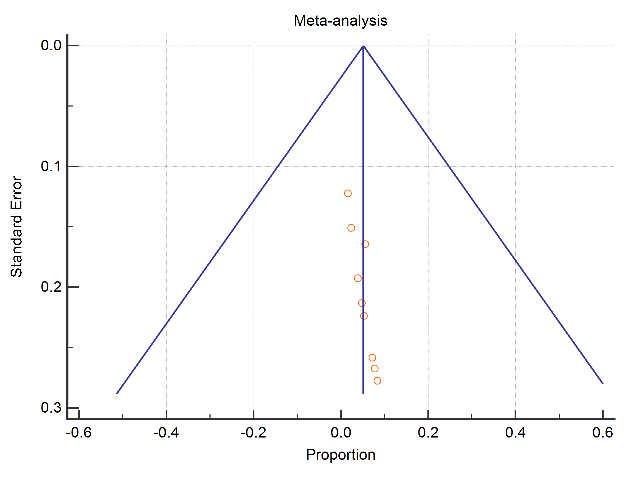** | 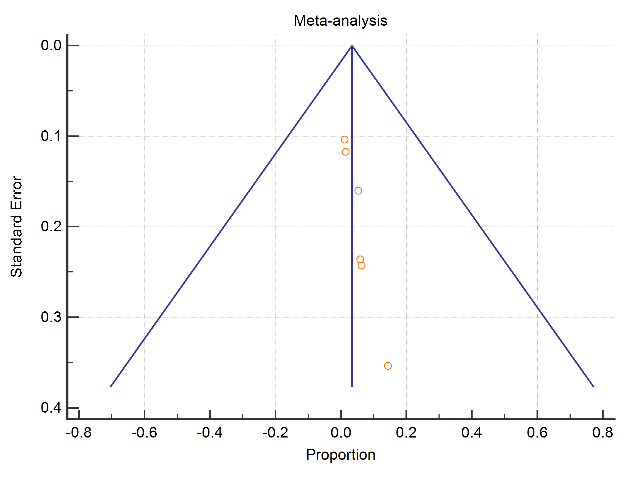 |
| **Nonunion** | 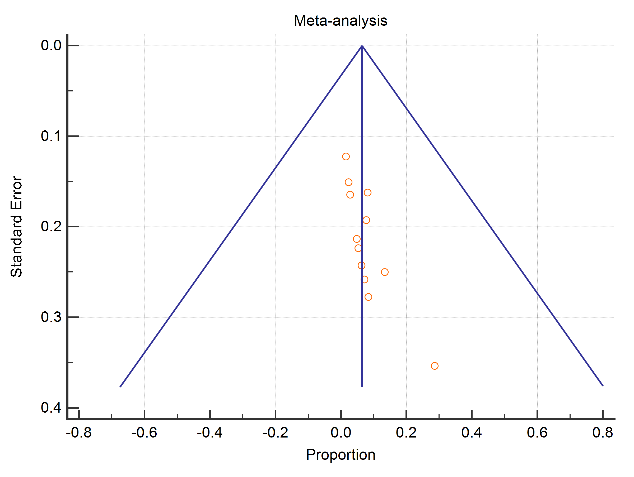 | 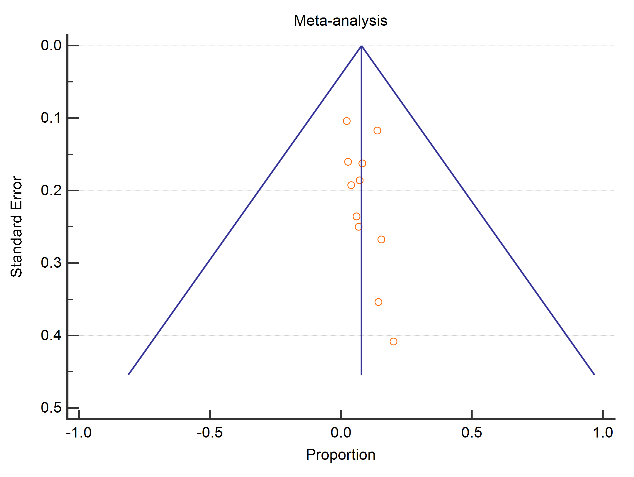 |
| **Varus malalignment** | 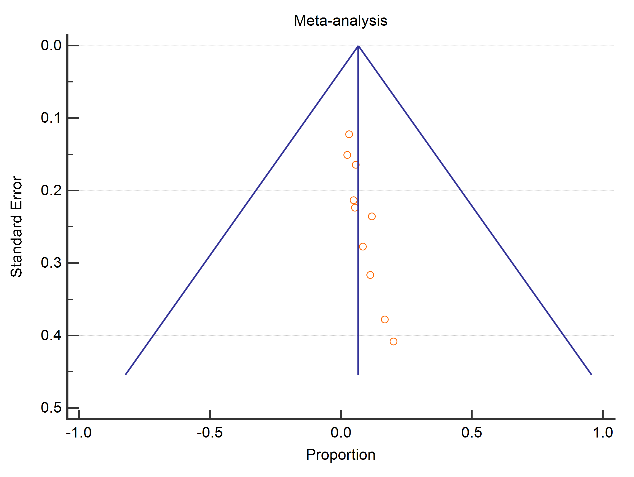 | 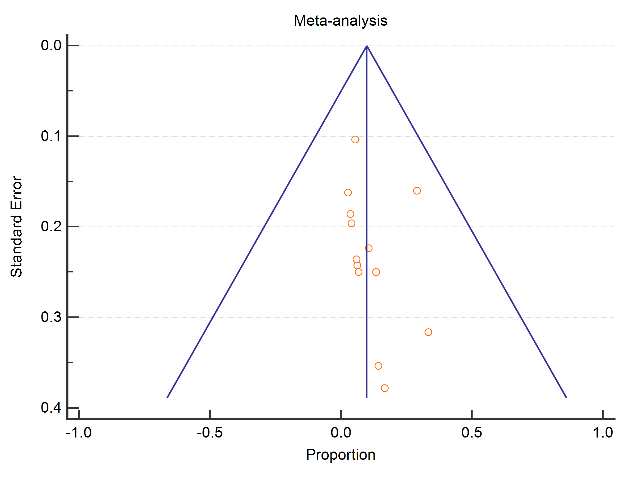 |

**Figure 7: Funnel plots of the pooled analyses of complications of the femur shaft, funnel plots for single implant are displayed and the left and for double implants on the right side.**

|  | **Single implant** | **Double implant** |
| --- | --- | --- |
| **Postoperative infection** | **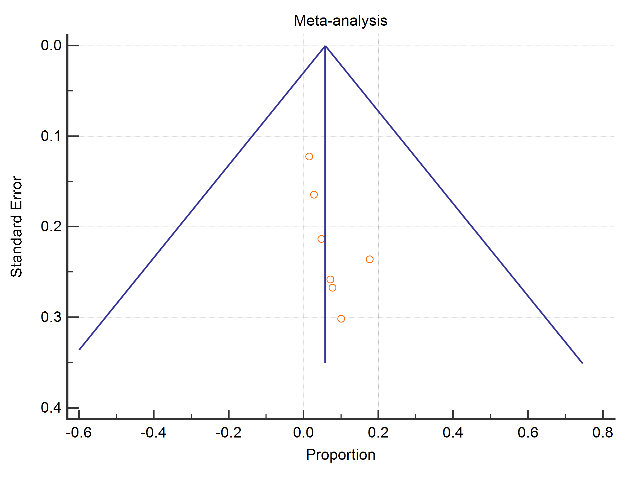** | 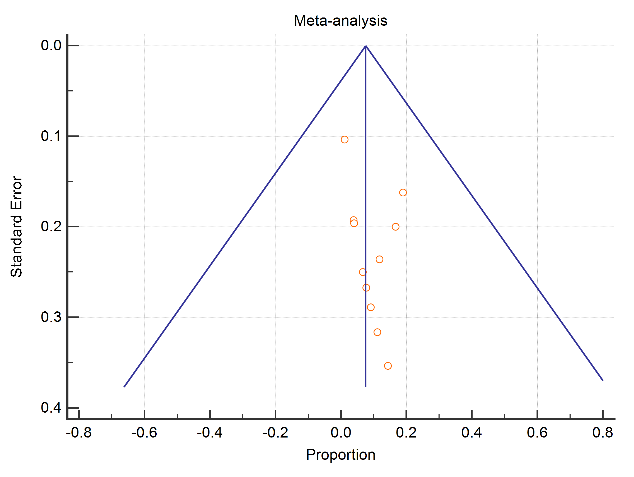 |
| **Delayed union** | 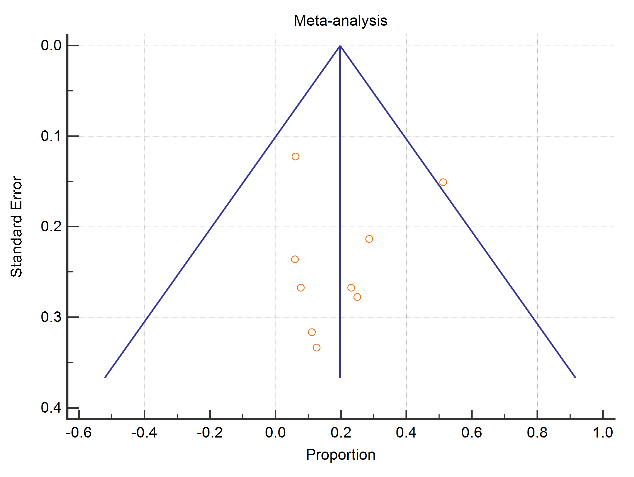 | **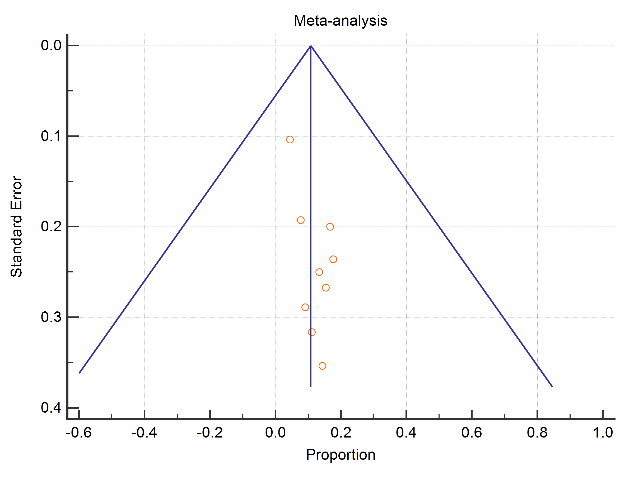** |
| **Nonunion** | 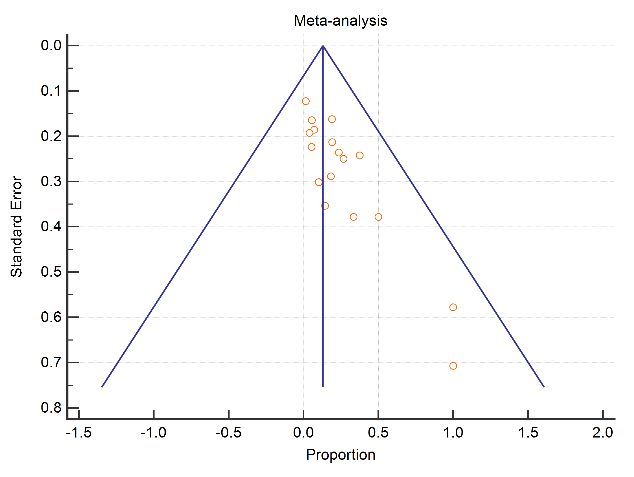 | 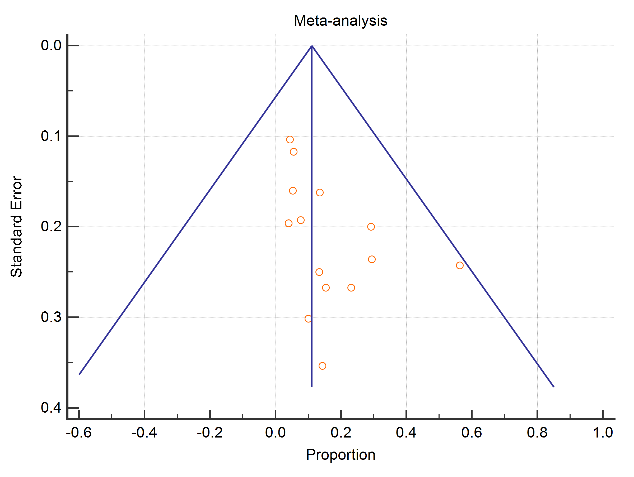 |
| **Malunion** | 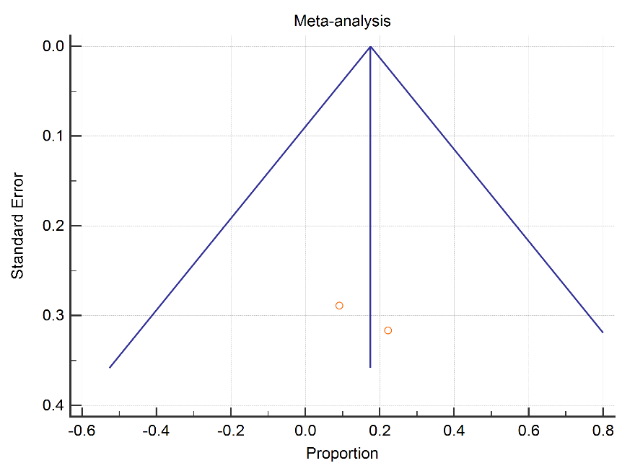 | 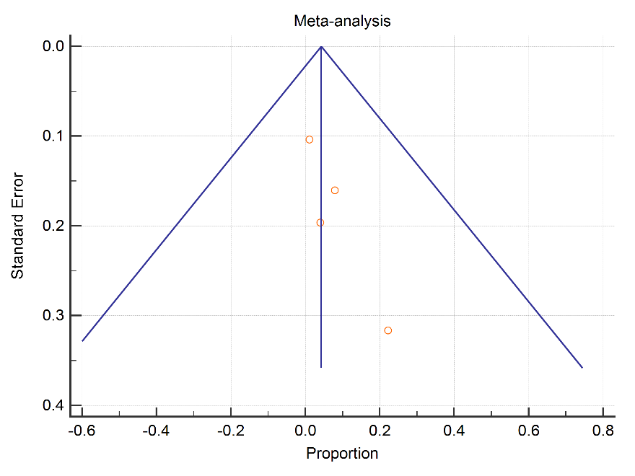 |

**Figure 8: Funnel plots of the pooled analyses of revision surgery. funnel plots for single implant are displayed and the left and for double implants on the right side.**

|  | **Single implant** | **Double implant** |
| --- | --- | --- |
| **Hardware failure** | 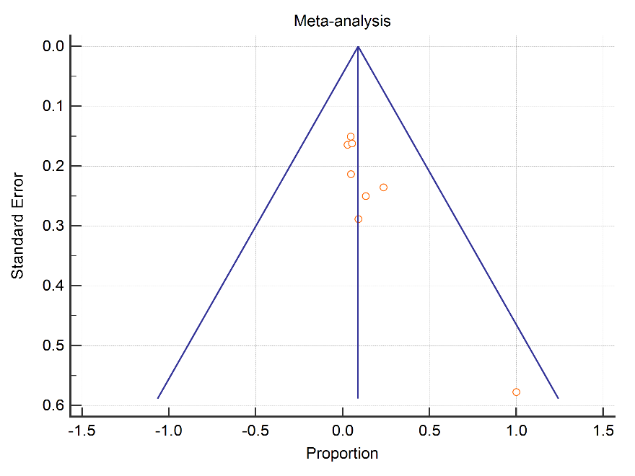 | 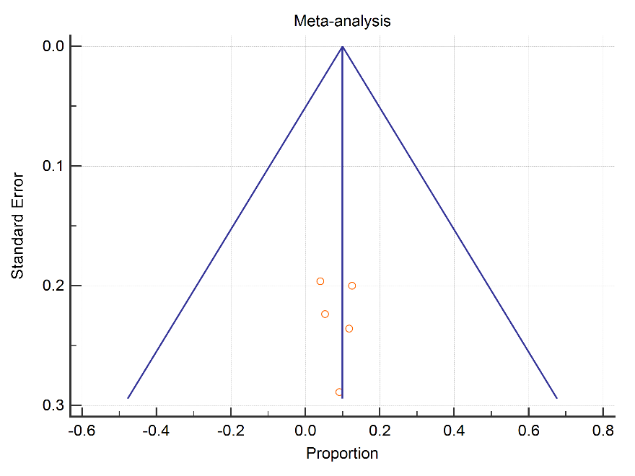 |
| **Revision surgery** | 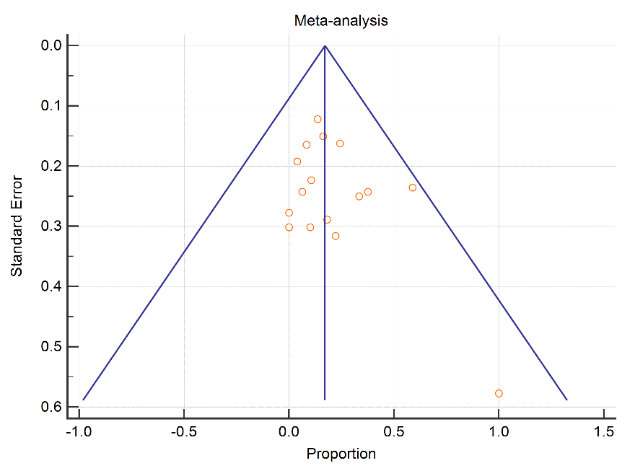 | 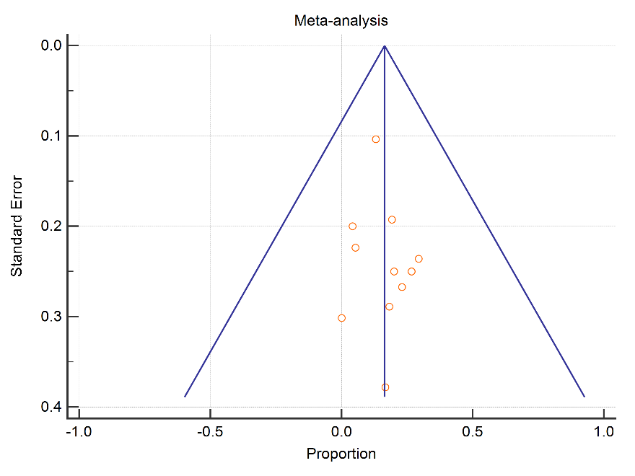 |

**Figure 9: Funnel plots of the pooled analyses of functional outcome. funnel plots for single implant are displayed and the left and for double implants on the right side.**

|  | **Single implant** | **Double implant** |
| --- | --- | --- |
| **Leg length discrepancy** | 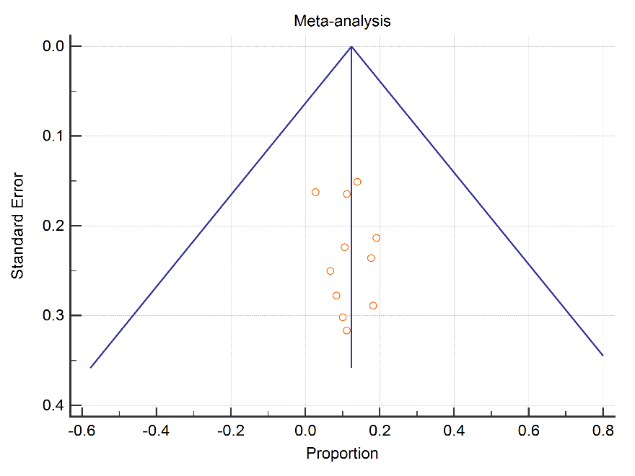 | 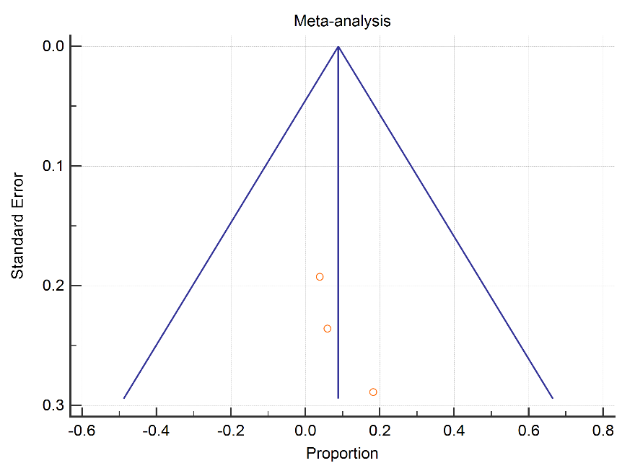 |
| **Functional outcome** | 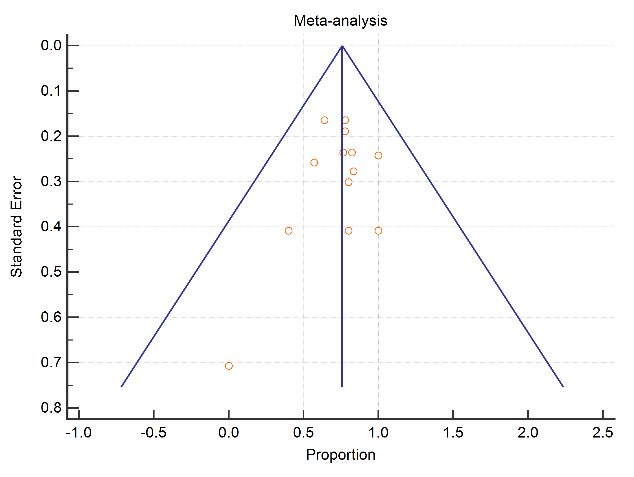 | 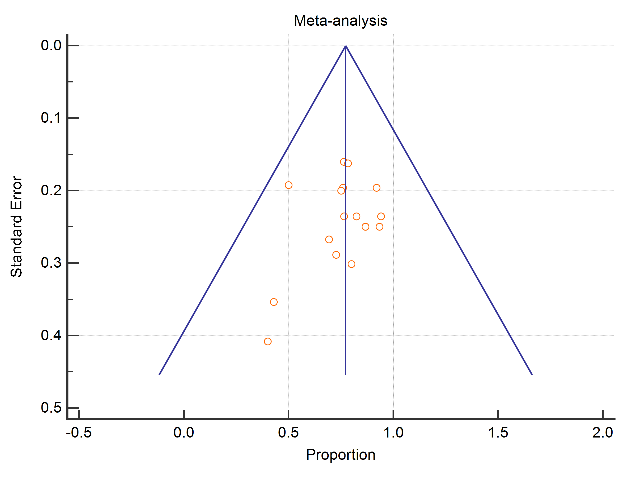 |
